# Supplementary figures and images for: Biocompatibility and Degradation Behavior of Molybdenum in an In Vivo Rat Model
Source: Materials (Basel). 2021 Dec 16;14(24):7776. doi: 10.3390/ma14247776 (PMC8705131; doi:10.3390/ma14247776)

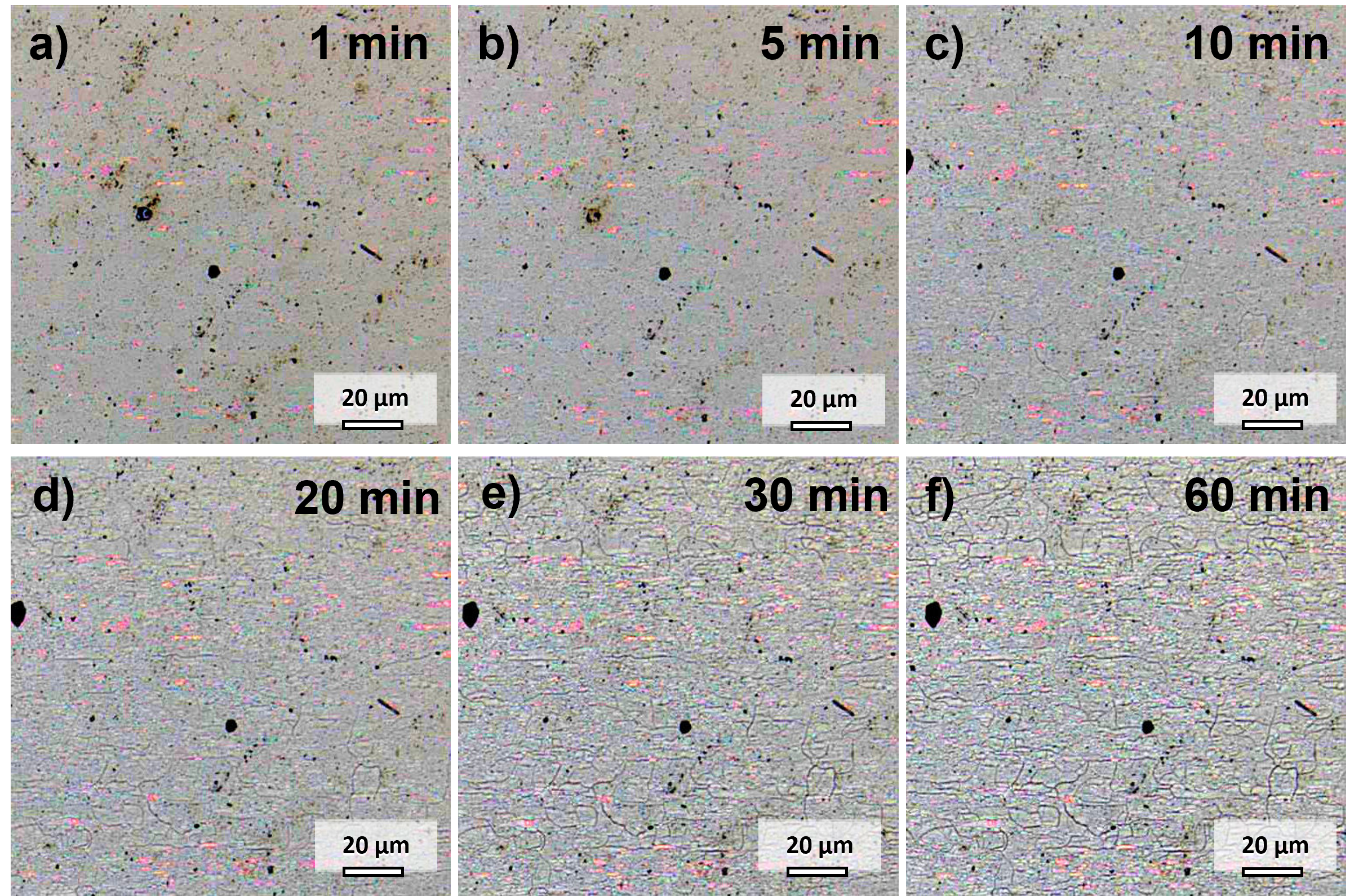

Supplement: Supplementary file 1 [file materials-14-07776-s001.zip › supplement/Figure S1.png]

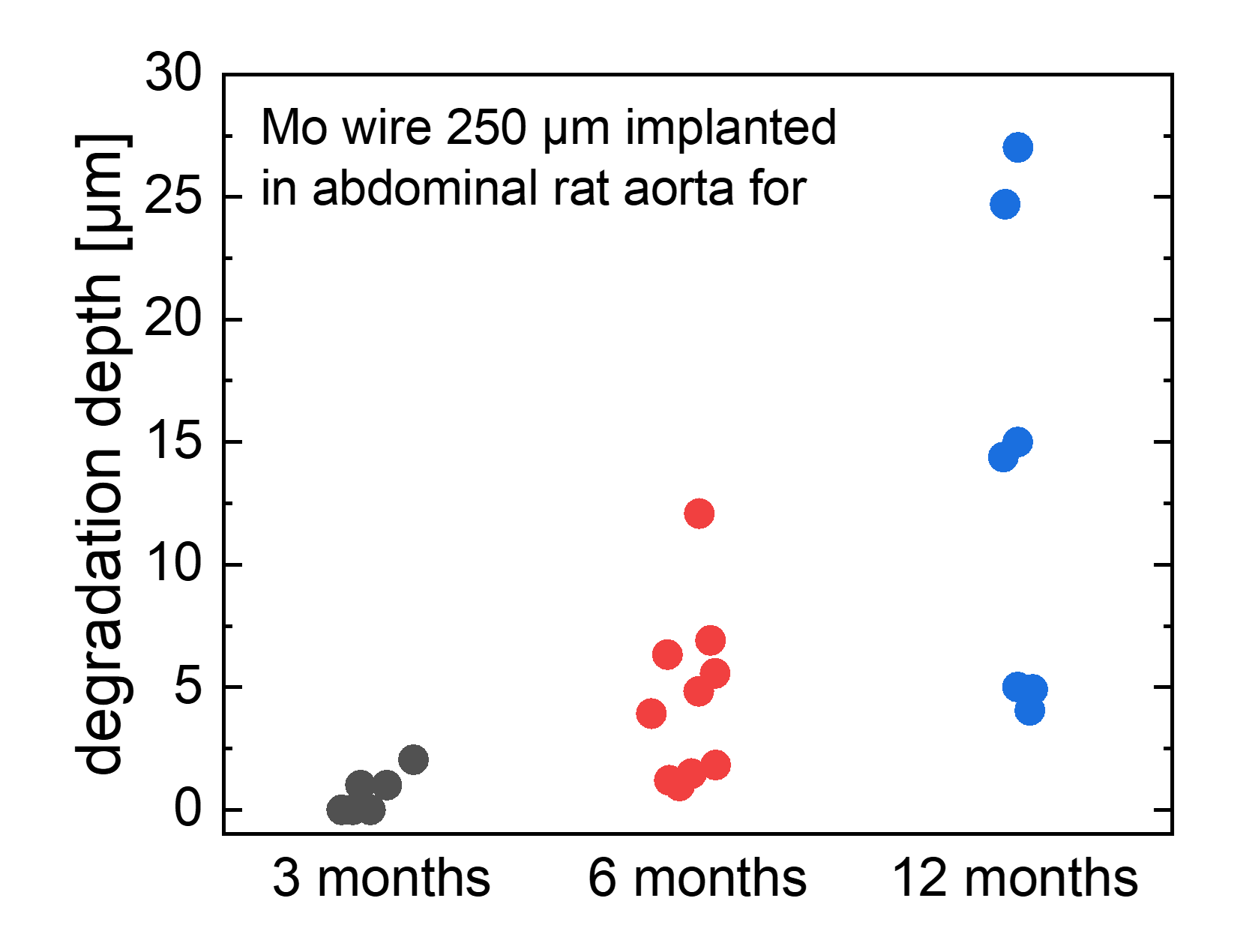

Supplement: Supplementary file 1 [file materials-14-07776-s001.zip › supplement/Figure S2.png]

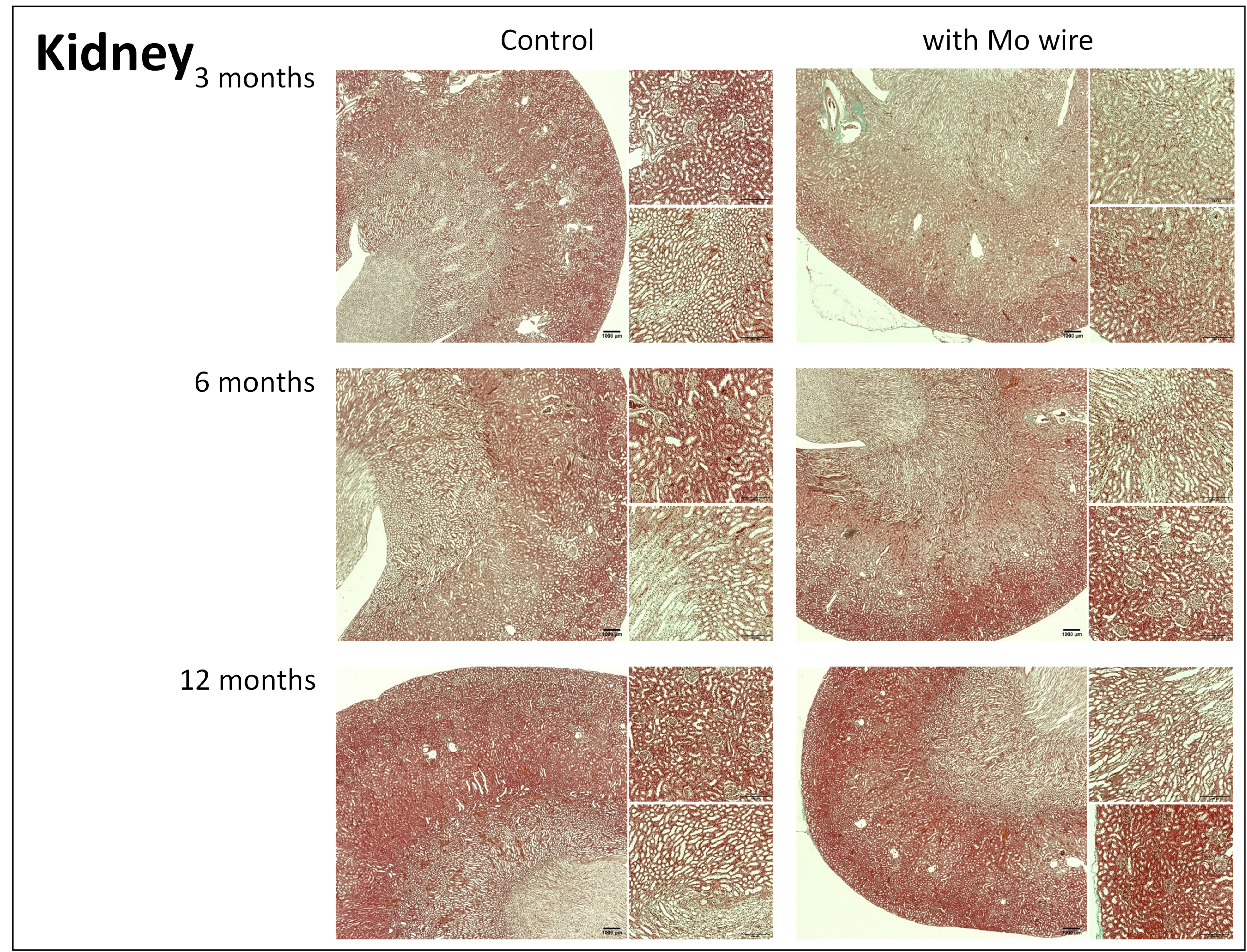

Supplement: Supplementary file 1 [file materials-14-07776-s001.zip › supplement/Figure S3.png]

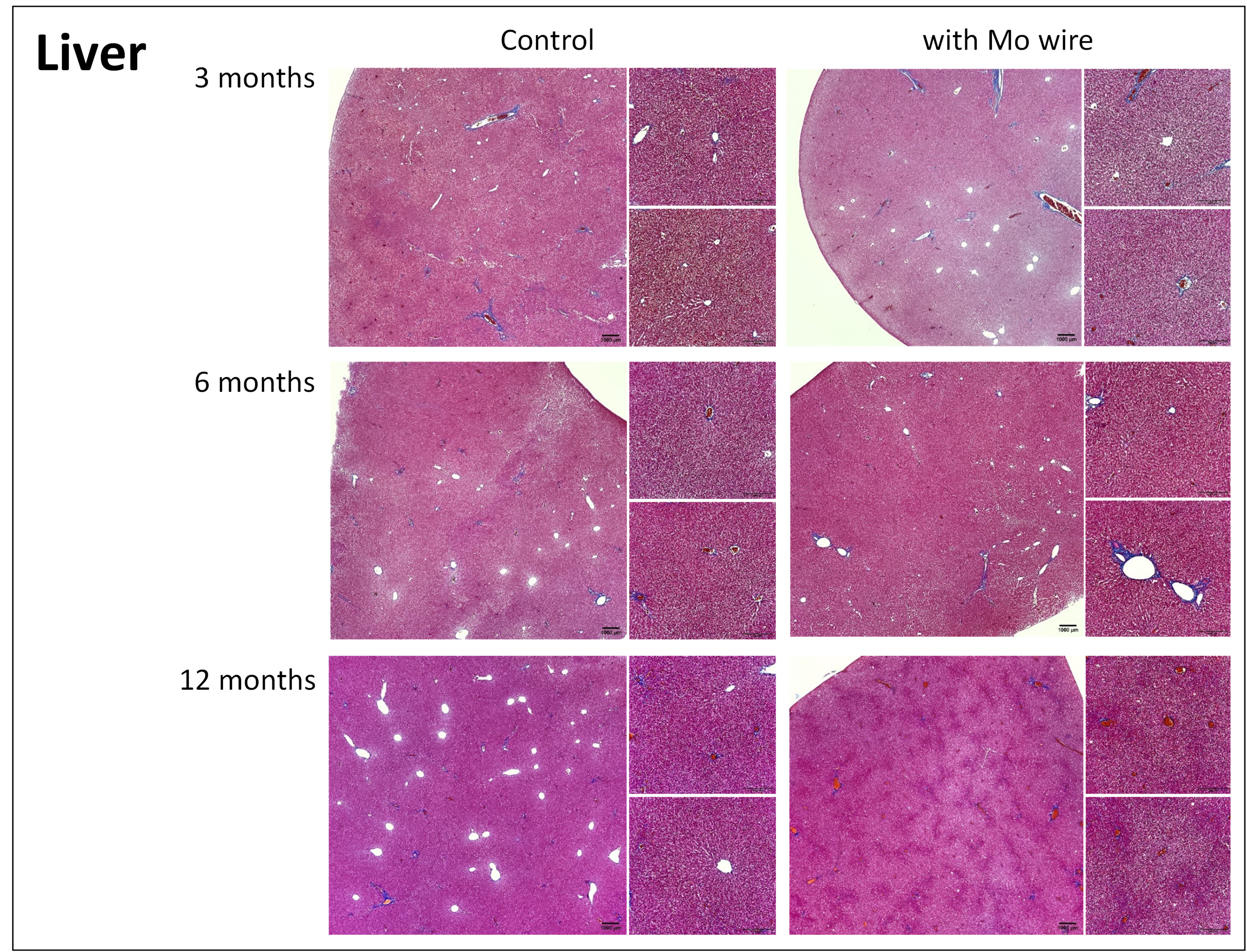

Supplement: Supplementary file 1 [file materials-14-07776-s001.zip › supplement/Figure S4.png]

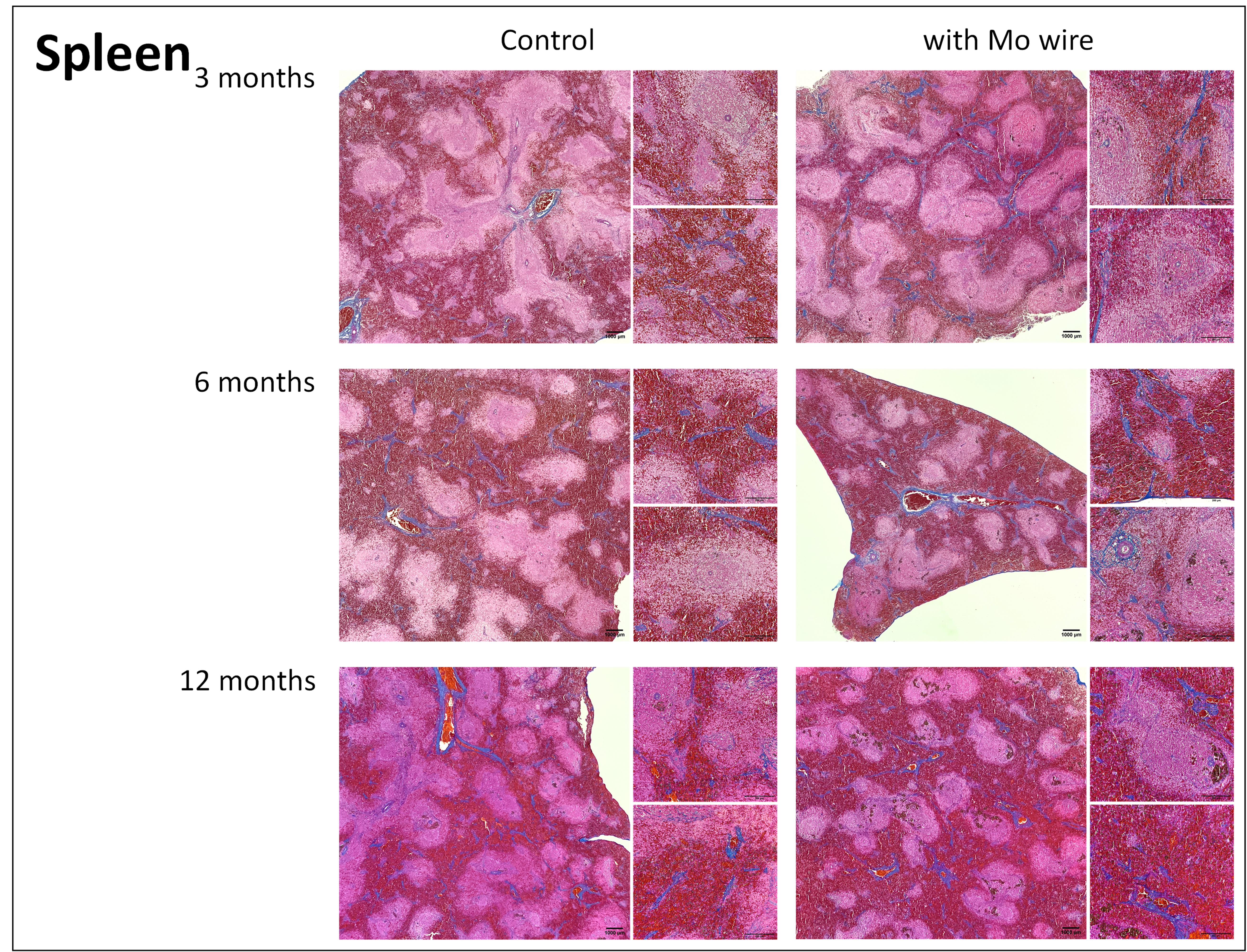

Supplement: Supplementary file 1 [file materials-14-07776-s001.zip › supplement/Figure S5.png]

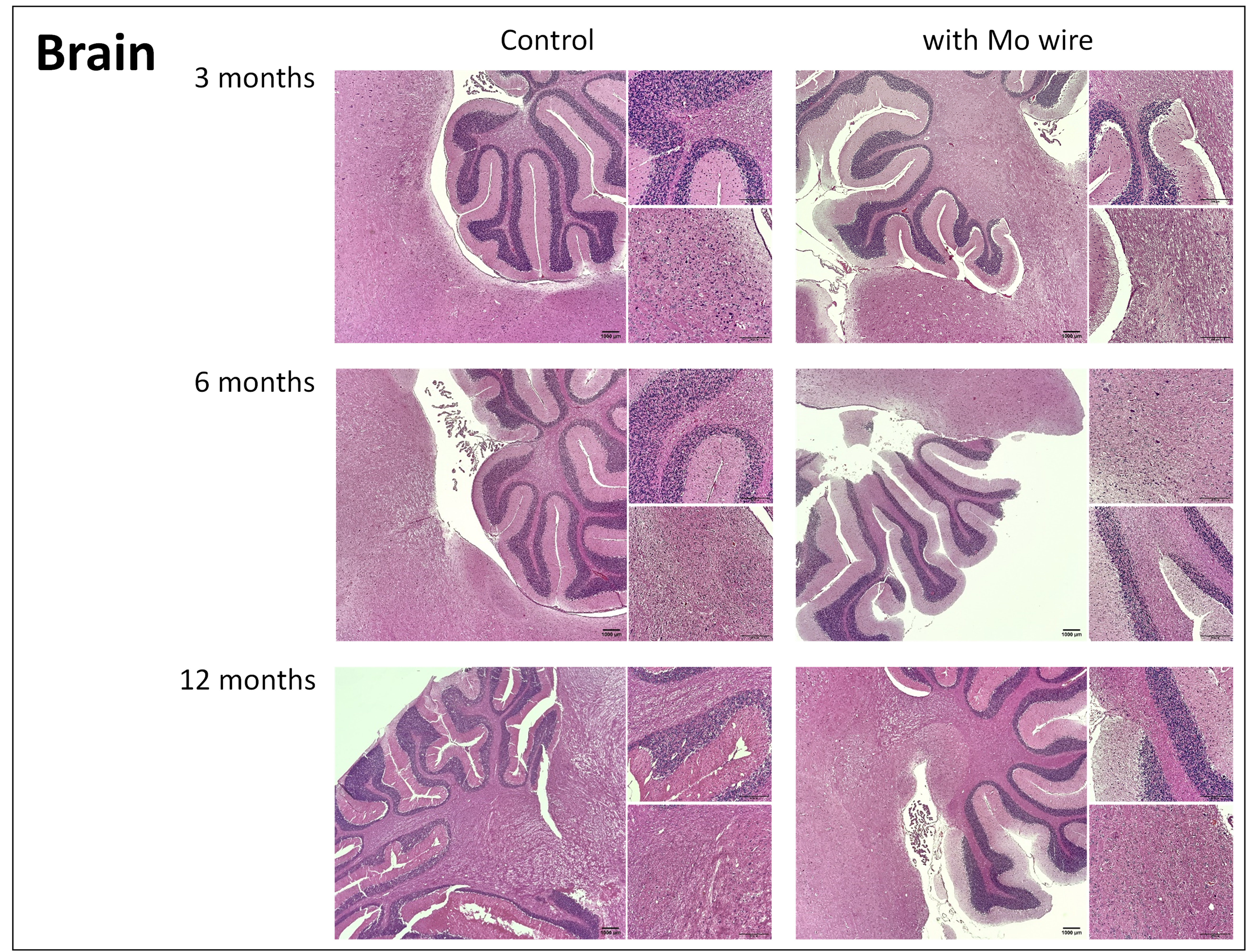

Supplement: Supplementary file 1 [file materials-14-07776-s001.zip › supplement/Figure S6.png]

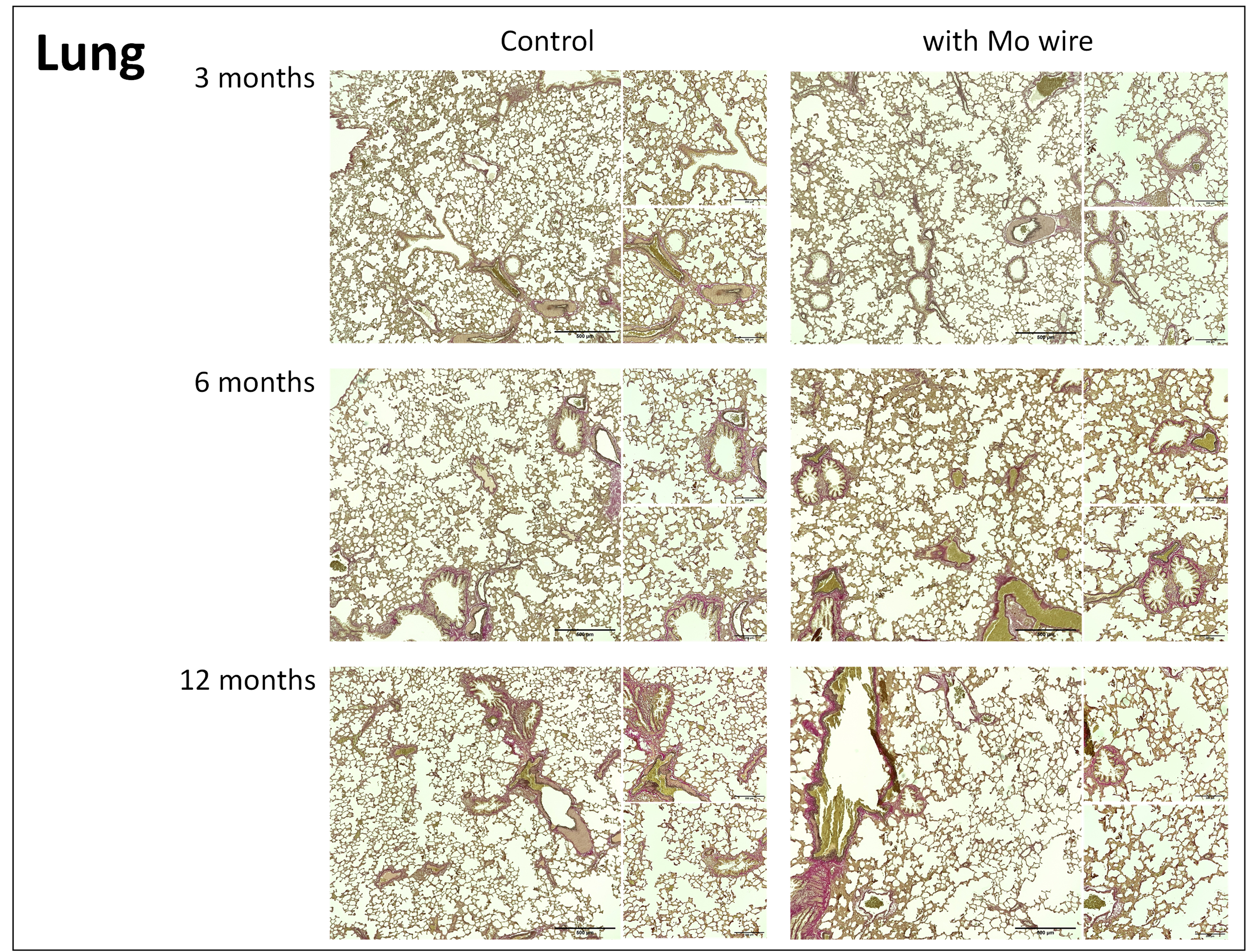

Supplement: Supplementary file 1 [file materials-14-07776-s001.zip › supplement/Figure S7.png]
